# Comparative efficacy of holmium laser versus plasma techniques in the surgical management of non-muscle invasive bladder cancer: a meta-analysis

**DOI:** 10.3389/fonc.2025.1625901

**Published:** 2025-08-06

**Authors:** Dong Li, Jia-Neng Xu, Han-Kai Chen, Qiang Ren, Yu-Min Li

**Affiliations:** Department of Urology, Jiashan First People’s Hospital, Jiaxing, Zhejiang, China

**Keywords:** non-muscle invasive bladder cancer, holmium laser, plasma techniques, metaanalysis, surgical outcomes

## Abstract

**Background:**

This systematic review and meta-analysis compared the efficacy of Holmium Laser and Plasma Techniques in treating non-muscle invasive bladder cancer (NMIBC), focusing on surgical duration, safety, and tumor recurrence.

**Methods:**

Adhering to PRISMA guidelines, we included randomized controlled trials that directly compared Holmium Laser and Plasma Techniques in NMIBC treatment. The Cochrane Collaboration’s tool assessed study quality, and statistical analyses were conducted using fixed-effect or random-effects models based on heterogeneity. Sensitivity analyses and publication bias assessments were also performed.

**Results:**

Our search yielded 1158 potentially relevant articles, with 8 studies meeting our inclusion criteria. The analysis showed no significant difference in surgical duration between the two techniques. However, the Holmium Laser was associated with a significantly lower incidence of intraoperative bladder perforation (RR=0.10, P<0.001) and a reduced short-term tumor recurrence rate (RR=0.65, P<0.01). Sensitivity analysis confirmed the robustness of these findings, and no significant publication bias was detected.

**Conclusions:**

Holmium Laser provides safer outcomes and better efficacy in reducing postoperative tumor recurrence compared to Plasma Techniques in NMIBC management.

## 1 Introduction

Non-muscle invasive bladder cancer (NMIBC) represents a significant proportion of bladder cancer cases, characterized by the absence of tumor invasion into the muscularis propria of the bladder wall. The management of NMIBC is pivotal not only for immediate treatment success but also for long-term outcomes, given the disease’s propensity for recurrence and progression. Among the array of surgical interventions available, holmium laser and plasma techniques have emerged as notable modalities, each offering a unique set of advantages and potential limitations in the surgical management of NMIBC (1, 2). Holmium laser, a versatile tool in urology, is renowned for its precision and efficacy in tissue ablation and incision. Its application in NMIBC treatment, particularly in transurethral resection of bladder tumors (TURBT), has been associated with reduced bleeding, shorter hospital stays, and potentially lower recurrence rates. The laser’s high-energy pulses allow for effective tumor removal while minimizing damage to surrounding tissues, making it an attractive option for clinicians and patients alike (3, 4).

On the other hand, plasma techniques, which encompass technologies such as plasma kinetic resection and bipolar plasma vaporization, have also gained traction in NMIBC management. These techniques leverage the unique properties of plasma to achieve efficient and controlled tissue removal with minimal thermal spread. This can translate to reduced risks of obturator nerve reflex and collateral damage, which are critical considerations in bladder cancer surgery. The plasma approach is appreciated for its ability to maintain hemostasis and visibility during the procedure, thereby enhancing surgical precision and patient safety (5, 6). Despite the apparent benefits of both holmium laser and plasma techniques, the literature presents a spectrum of outcomes regarding their comparative efficacy in NMIBC treatment. Variability in study designs, patient populations, and outcome measures has contributed to conflicting reports on aspects such as recurrence rates, progression, operative time, and complications. This heterogeneity underscores the need for a systematic review and meta-analysis to synthesize available evidence and provide a clearer understanding of how these modalities compare in the context of NMIBC surgical management.

The objective of this systematic review and meta-analysis is to rigorously evaluate and compare the efficacy and safety of holmium laser versus plasma techniques in the treatment of NMIBC. By integrating data from diverse studies, this analysis aims to offer a comprehensive assessment that can inform clinical decision-making and guide future research directions.

## 2 Methods

### 2.1 Data extraction process for meta-analysis

In conducting our meta-analysis, we adhered to the stringent guidelines set forth by the Preferred Reporting Items for Systematic Reviews and Meta-Analyses (PRISMA) (7). To compile a comprehensive collection of relevant studies, we initiated a search across five major electronic databases: PubMed, Embase, Web of Science, Cochrane Library, and Wanfang Data. This search was conducted on September 19, 2023, and was characterized by the absence of temporal constraints to maximize the breadth of our data pool. Our search strategy was meticulously crafted, incorporating a series of key terms including “bladder cancer,” “bladder tumor,” “non-muscle-invasive bladder cancer,” “Holmium Laser,” “YAG,” and “plasmakinetic resection.” These terms were thoughtfully chosen to align with the PICO framework, which encompasses Patient, Intervention, Comparison, and Outcome, ensuring a thorough and inclusive retrieval of pertinent studies. We imposed no restrictions based on language to further widen the scope of our search. Additionally, we undertook a manual examination of the reference lists from relevant articles to identify any further studies that might contribute valuable data to our meta-analysis.

### 2.2 Data extraction process for meta-analysis

*Inclusion Criteria:*

1. Study Design: We included randomized controlled trials (RCTs) that directly compared the efficacy and safety of holmium laser and plasma techniques in the treatment of NMIBC.
2. Participants: Studies involving patients diagnosed with NMIBC, irrespective of age, gender, or ethnicity, were considered.
3. Interventions: Only studies that employed holmium laser or plasma techniques as the primary surgical intervention for NMIBC were included.
4. Comparators: Studies must have compared holmium laser and plasma techniques directly within the same study to be considered for inclusion.
5. Outcomes: Studies were required to report on at least one of the following outcomes: recurrence rates, progression rates, operative times, complication rates, or any other clinically relevant outcomes associated with the treatment of NMIBC.

*Exclusion Criteria:*

1. Study Design: Case reports, editorials, reviews, meta-analyses, and animal studies were excluded due to their inherent methodological differences and potential biases.
2. Participants: Studies focusing on muscle-invasive bladder cancer or other urological conditions unrelated to NMIBC were excluded.
3. Interventions: Studies that did not specifically employ holmium laser or plasma techniques as the intervention for NMIBC were excluded.
4. Outcomes: Studies lacking clear outcome measures relevant to the efficacy and safety of NMIBC treatment were not considered.
5. Incomplete Data: Studies with incomplete data or those that did not provide a direct comparison between holmium laser and plasma techniques were excluded.
6. Duplicate Data: In cases of multiple publications reporting on the same patient cohort, only the most comprehensive or recent report was included to avoid duplication of data.

### 2.3 Data extraction process for meta-analysis

During our meta-analysis, literature screening and data extraction were conducted with precision by two independent reviewers. They independently gathered essential information from each study, ensuring an unbiased and thorough review. Conflicts in their findings were resolved through discussion or, if necessary, by consulting a third party. Key information extracted included authors, publication year, and case numbers, alongside participant age, tumor size, and number. These details were vital for evaluating the relevance of each study and for a detailed comparison of treatment outcomes. For missing data, we proactively contacted original authors, seeking unpublished information to enrich our analysis.

### 2.4 Quality assessment

The assessment of study quality was conducted using the Cochrane Collaboration’s tool for evaluating the risk of bias (8). This evaluation was carried out independently by two reviewers who scrutinized several key areas, including the generation of random sequences, concealment of allocation, blinding of study participants and staff, the completeness of outcome data, the presence of selective outcome reporting, and the potential for other biases. For each of these areas, the risk of bias was categorized as low, unclear, or high. Any discrepancies in the assessments made by the reviewers were addressed through deliberation, and when needed, a third reviewer was consulted to achieve a consensus.

### 2.5 Statistical analyses

In our meta-analysis, statistical analyses were conducted to evaluate the heterogeneity across studies, initially assessed through chi-square statistics and further quantified by the I^2^ value. For cases where the I^2^ value was below 50% and the corresponding P-value was at least 0.10, indicating negligible heterogeneity, a fixed-effect model was utilized to calculate the combined effect size. Conversely, significant heterogeneity was inferred from an I^2^ value of 50% or higher, or a P-value below 0.10, prompting the use of a random-effects model for effect size computation. To ensure the robustness of our findings, sensitivity analysis was performed, systematically excluding each study to observe its impact on the overall effect size. This step was crucial for identifying any single study’s undue influence on the meta-analysis outcome. To address potential publication bias, the symmetry of the funnel plot was examined, with an equitable distribution suggesting minimal bias. Egger’s linear regression test provided a quantitative assessment of publication bias, complementing the visual inspection of the funnel plot. All statistical tests were two-sided, with a significance threshold set at a P-value less than 0.05. Stata version 17 was employed for all data analyses, ensuring a rigorous and comprehensive statistical evaluation of the included studies.

## 3 Results

### 3.1 Search results and study selection

**Figure 1:**
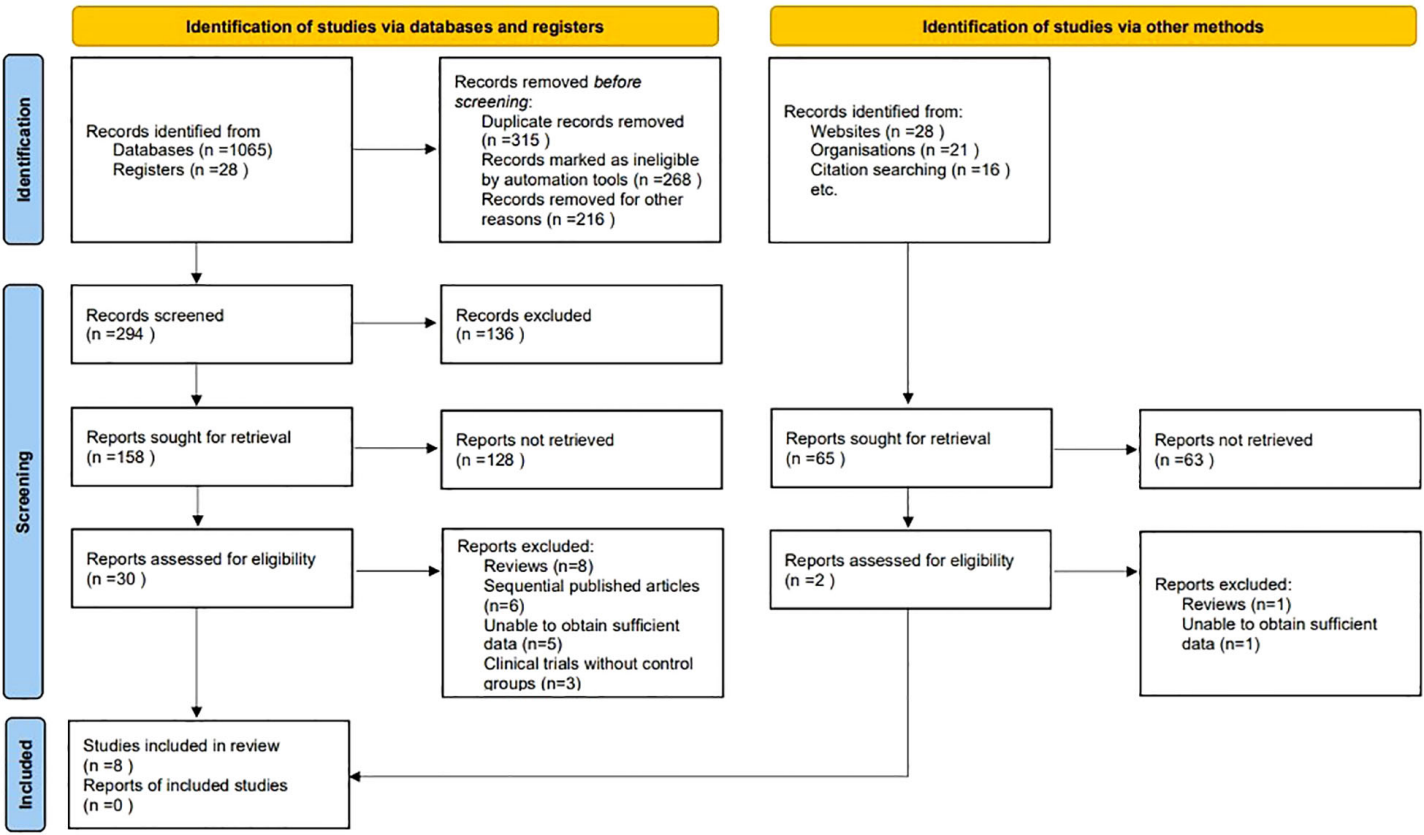
Flowchart depicting the study selection process for inclusion in the meta-analysis.

At the outset of our systematic review and meta-analysis, a comprehensive search across several electronic databases yielded an initial tally of 1158 potentially relevant articles. To streamline this collection, duplicates were eliminated through a systematic filtering process, ensuring the uniqueness of each study. Following this, titles and abstracts underwent a rigorous screening based on predefined inclusion and exclusion criteria, which encompassed study design, participant demographics, clinical outcomes, and research quality. This initial screening phase distilled the number of articles to 32, warranting a detailed full-text review. Conducted by multiple independent investigators, this review aimed to eliminate bias and ensure a thorough evaluation. Subsequent to this in-depth analysis, 24 articles were excluded due to various reasons: 9 were review articles, 6 were sequentially published works, 6 contained insufficient data, and 3 were clinical trials without control groups. Ultimately, 8 studies satisfied all the stringent criteria set forth in our protocol, making them eligible for inclusion in the final analysis (9–16) (**Figure 1**).

### 3.2 Study characteristics summary

The meta-analysis encompassed a diverse set of studies, each contributing unique insights into the comparative efficacy of holmium laser versus plasma techniques in the surgical management of bladder tumors. The included studies, published between 2015 and 2020, involved a total of 513 cases treated with Holmium Laser and an equal number treated with Plasma Techniques. The participant age across these studies ranged approximately from 50 to 64 years, indicating a middle-aged to elderly population typically affected by bladder tumors. A notable aspect of these studies was the variation in tumor characteristics and treatment outcomes reported. While some studies provided detailed measurements of tumor size, with averages ranging from 1.5 to 2.8 cm for Holmium Laser treatments and closely matching values for Plasma Techniques, other studies did not report this information. The distribution of single versus multiple tumors was also documented, highlighting the clinical scenarios under which each technique was applied, though not all studies reported these details. Gender distribution among participants showed a slight male predominance in most studies, reflecting the general epidemiology of bladder cancer (**Table 1**).

**Table 1:** Basic characteristics of included studies.

Author	Year	Age (H/P)	Male (H/P)	Cases (H/P)	Tumor Size (H/P)	Single tumor (H/P)	Multiple tumors (H/P)
Huang Jianhong (11)	2018	52.49 ± 11.26/53.08 ± 10.77	27/26	50/50	NA	34/37	16/13
Huang Zhongli (16)	2015	62.6 ± 11.3/61.6 ± 11.5	33/40	50/50	1.5 ± 0.9/1.5 ± 0.8	33/36	17/14
Jiang Shuai (12)	2016	60/60	4/5	56/55	NA	45/42	15/18
Li Wei (13)	2017	62.36 ± 9.26/61.82 ± 9.2	28/24	43/43	1.62 ± 0.64/1.68 ± 0.6	NA	NA
Shen Hongfeng (10)	2017	54.5 ± 5.7/55.2 ± 6.0	122/113	180/180	1.5 ± 0.7/1.6 ± 0.6	145/152	35/27
Sun Yuansheng (15)	2019	64.44 ± 12.45/61.34 ± 13.1	35/40	52/53	NA	37/31	15/22
Zhang Heqing (9)	2020	50.51 ± 12.28/50.62 ± 12.3	20/21	34/34	2.26 ± 1.54/2.32 ± 1.48	24/23	10/11
Zhang Wenzhi (14)	2020	54.9 ± 9.23/56.3 ± 8.91	42/40	48/48	2.8 ± 1.17/2.9 ± 1.12	39/36	9/12

NA, Not available; H/P, Holmium Laser/Plasma Techniques.

### 3.3 Results of quality assessment

**Figure 2:**
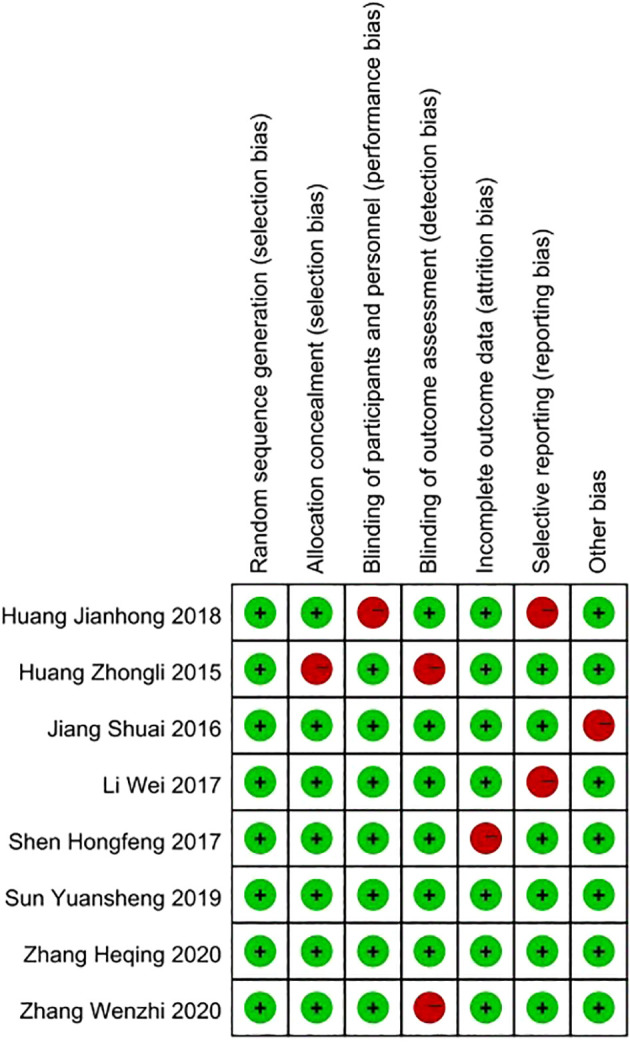
Quality assessment of included studies using Cochrane Collaboration’s tool criteria. Red in figure indicates high risk, and green means low risk.

The quality assessment of the included studies, following the Cochrane Collaboration’s risk of bias tool, revealed a spectrum of methodological strengths and weaknesses. Overall, the majority of studies demonstrated a low risk of bias in random sequence generation and allocation concealment, indicating a robust approach to randomization and the maintenance of allocation secrecy. Concerning the blinding of participants and personnel, the studies showed mixed results, with several studies achieving low risk, demonstrating that participant and personnel were appropriately blinded to the intervention. However, there were instances where the blinding was unclear or not carried out, suggesting potential performance bias in those studies. The assessment of outcome data indicated that the majority of studies had a low risk of incomplete outcome data, suggesting that the results reported were generally complete and likely reliable. Selective reporting bias was predominantly low across studies, indicating that the reported findings were pre-specified and consistently delivered. The domain of other bias’s was also well addressed, with most studies showing a low risk, which implies a general absence of additional factors that could unduly influence the studies’ outcomes. In conclusion, while the overall quality of the studies was commendable, some areas, particularly pertaining to blinding, did present variability, which should be considered when interpreting the results of this meta-analysis (**Figure 2**).

### 3.4 Surgical duration analysis

**Figure 3:**
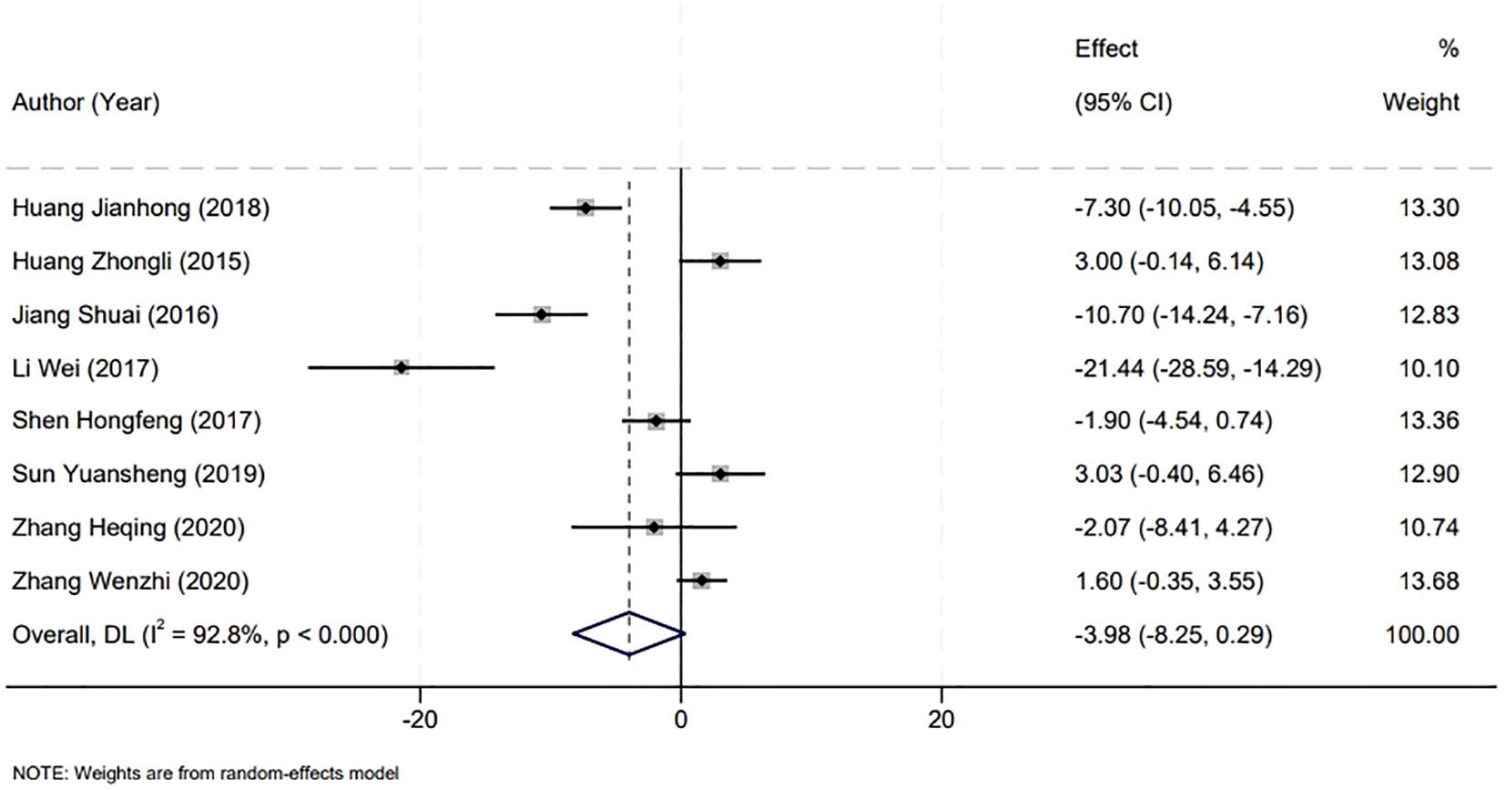
Forest plot showing the comparison of surgical duration between groups.

The analysis of surgical duration was a critical component of our meta-analysis, which included data from eight studies that reported on operation times. A significant heterogeneity was observed among the included studies (P<0.001, I^2^ = 92.8%), necessitating the use of a random-effects model for the analysis. Upon examination, the comparison between the Holmium Laser group and the Plasma group revealed no statistically significant difference in the duration of surgery. The weighted mean difference (WMD) was -3.98 minutes, with a 95% confidence interval (CI) ranging from -8.25 to 0.29, and a P-value greater than 0.05. This suggests that the choice between Holmium Laser and Plasma techniques for the surgical management of non-muscle invasive bladder cancer may not be influenced by the factor of operative time, as both modalities demonstrate similar durations in the surgical setting (**Figure 3**).

### 3.5 Incidence of bladder perforation during surgery

**Figure 4:**
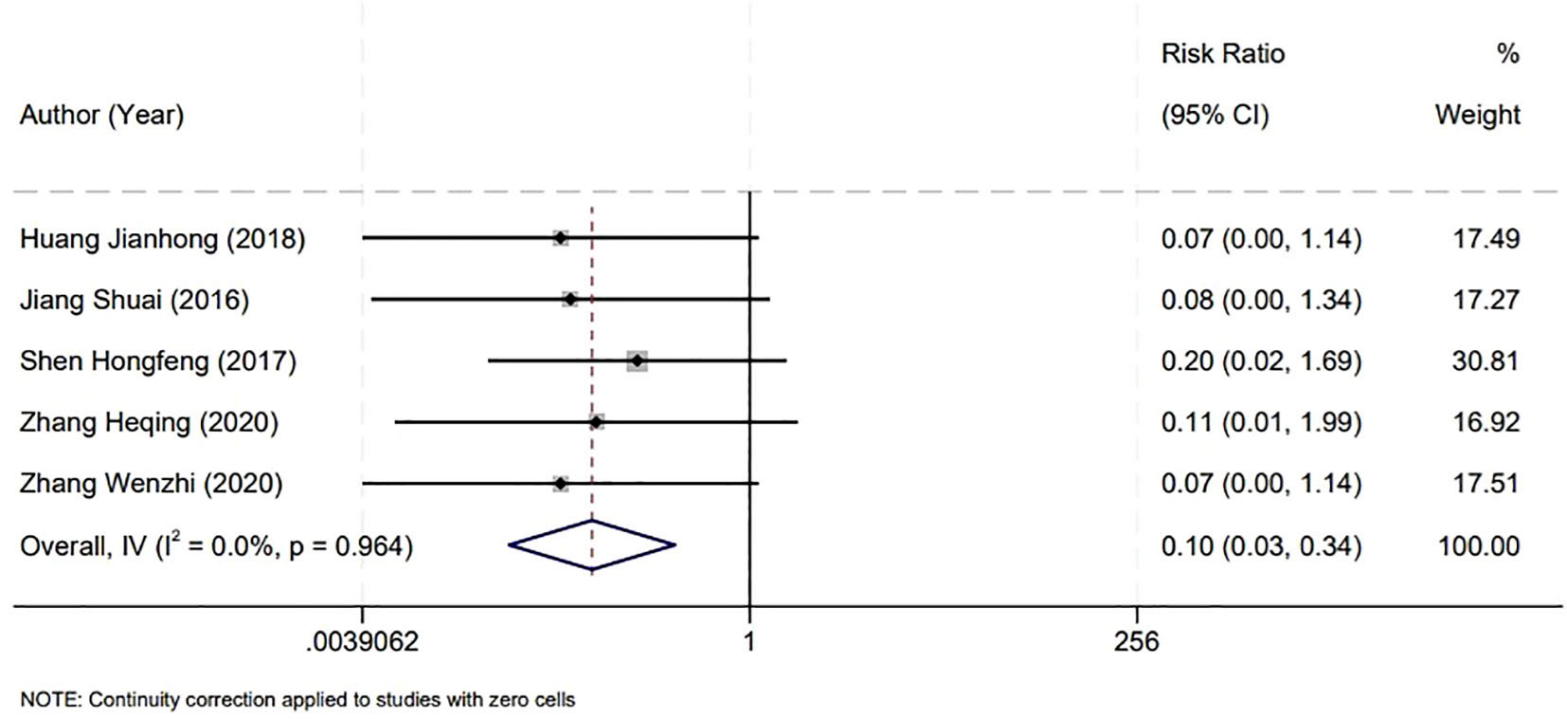
Forest plot illustrating the comparison of bladder perforation rates during surgery.

In assessing the safety outcomes of bladder surgeries, our meta-analysis focused on the incidence of intraoperative bladder perforation, incorporating findings from five studies. The analysis revealed no significant heterogeneity among these studies (P=0.964, I^2^ = 0.0%), which supported the use of a fixed-effect model for statistical analysis. The results indicated a notably lower rate of bladder perforation in the Holmium Laser group compared to the Plasma group. This difference was statistically significant, with a relative risk (RR) of 0.10 and a 95% CI from 0.03 to 0.34, and a P-value of less than 0.001. This significant discrepancy underscores the potential advantages of the Holmium Laser technique in minimizing the risk of bladder perforation during surgical procedures (**Figure 4**).

### 3.6 Postoperative tumor recurrence rate

**Figure 5:**
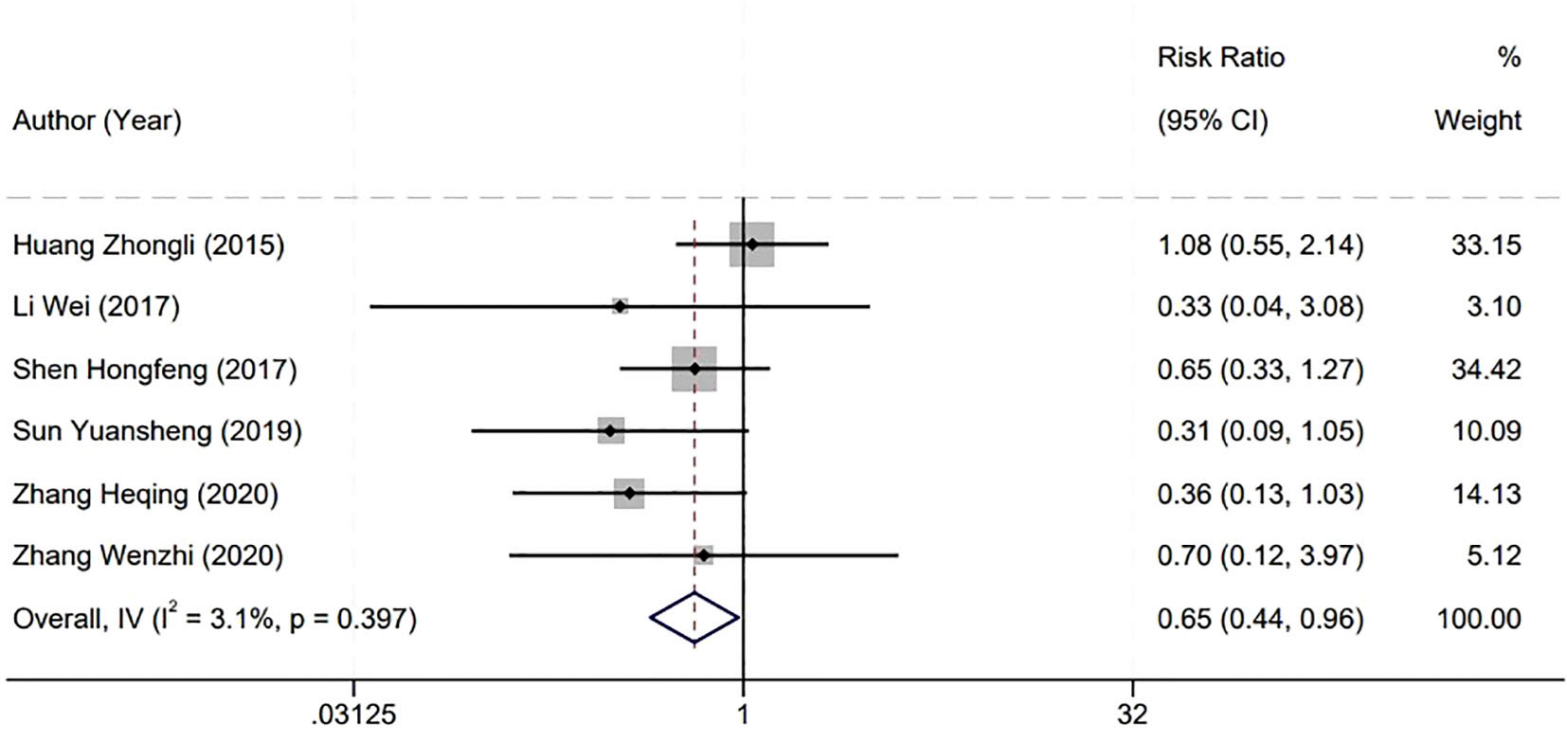
Forest plot presenting the comparison of postoperative tumor recurrence rates.

In evaluating the efficacy of surgical interventions for bladder tumors, our meta-analysis specifically examined the postoperative short-term tumor recurrence rate, drawing from six studies. The statistical homogeneity observed among these studies (P=0.397, I^2^ = 3.1%) warranted the application of a fixed-effect model for analysis. The analysis revealed a lower short-term recurrence rate of bladder tumors in the Holmium Laser group compared to the Plasma group, with this difference reaching statistical significance. The RR stood at 0.65 with a 95% CI spanning from 0.44 to 0.96, and the P-value was less than 0.01. This outcome suggests a potential benefit of the Holmium Laser technique in reducing the incidence of early tumor recurrence following surgery. The statistical significance of this finding underscores its potential impact on clinical decision-making and patient care strategies, particularly when considering the long-term management and surveillance of patients after bladder tumor resection (**Figure 5**).

### 3.7 Sensitivity analysis to evaluate result consistency

**Figure 6:**
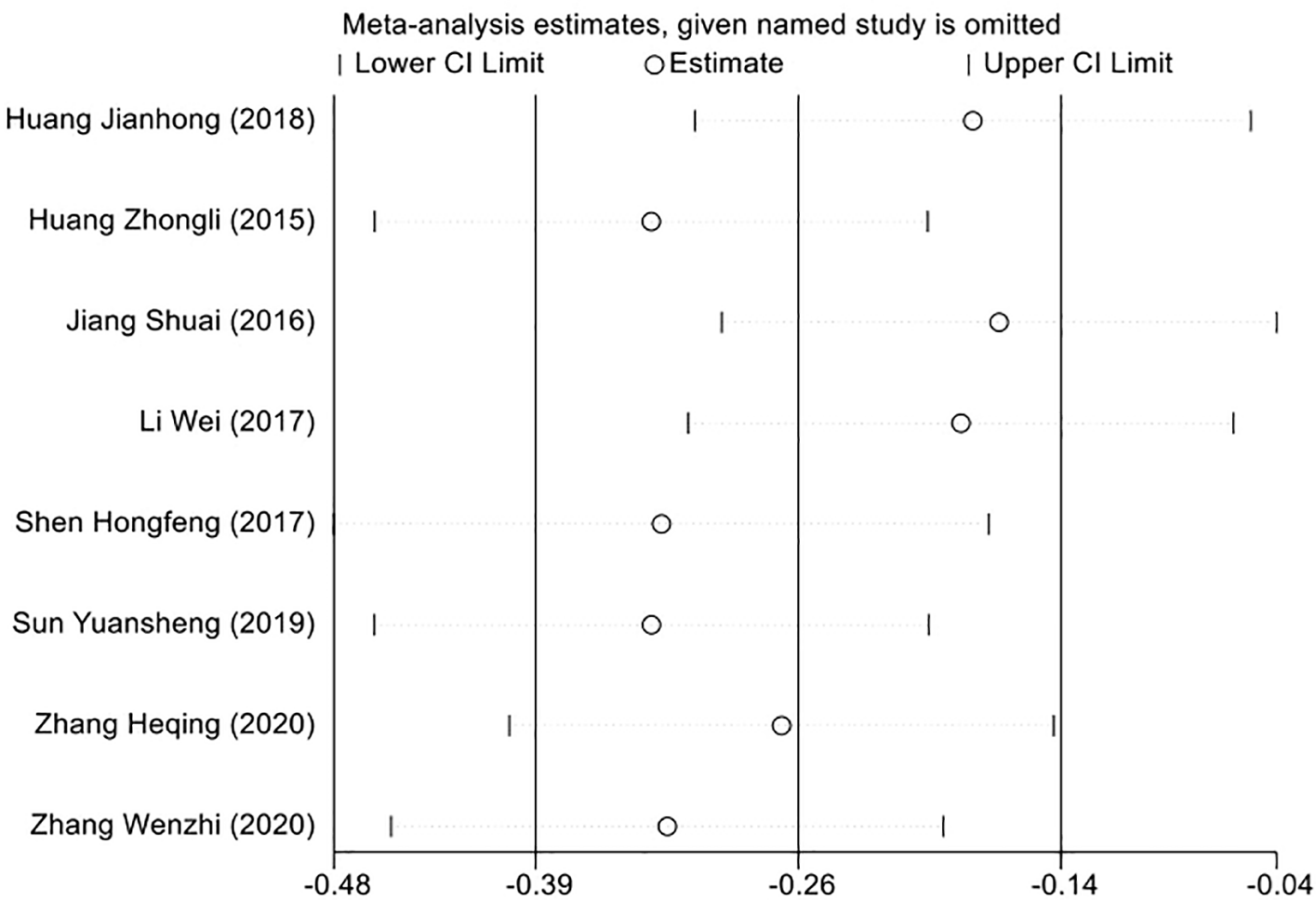
Sensitivity analysis graph to verify the consistency of the meta-analysis findings.

To ensure the robustness of our meta-analysis findings, we employed a sensitivity analysis in light of the heterogeneity identified across the included studies. This involved a leave-one-out approach, where we recalculated the overall effect size after sequentially removing each study. Through this method, we aimed to determine the influence of each individual study on the aggregate results. The outcome of the sensitivity analysis demonstrated that the pooled effect size was consistent, with no single study disproportionately affecting the overall results. This consistency supports the conclusion that the findings of our meta-analysis are not reliant on any specific study, but rather reflect a generalizable effect across the studies included (**Figure 6**).

### 3.8 Assessment of publication bias in meta-analysis

**Figure 7:**
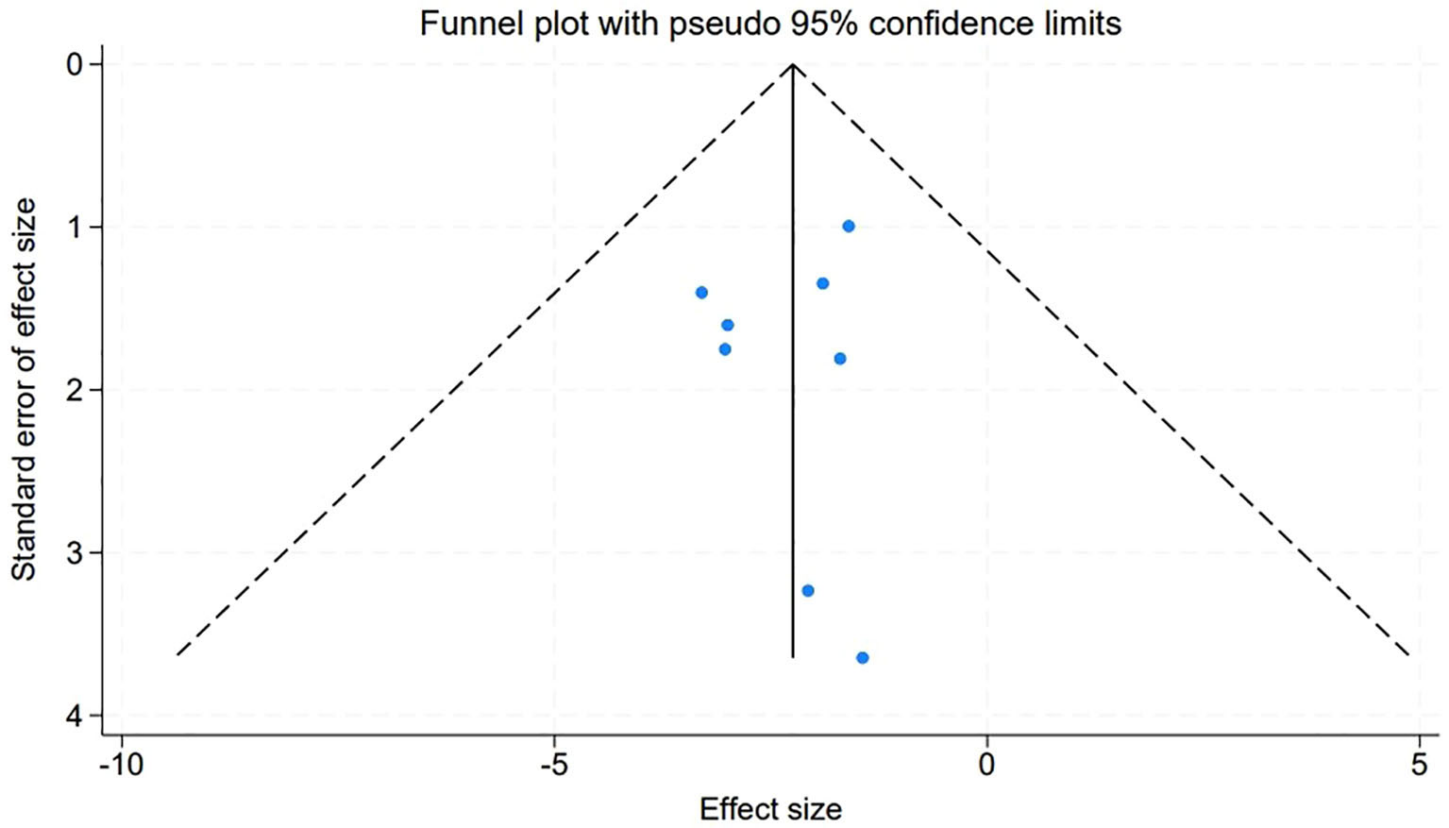
Funnel diagram for assessing the potential publication bias in the included studies.

An evaluation of publication bias within our meta-analysis was meticulously conducted through the construction of funnel plots for the studies included. The symmetry observed in these plots suggests an absence of publication bias, with the visual representation depicted in **Figure 7** corroborating this finding. Further quantitative confirmation was provided by Egger’s linear regression test, which was applied to various variables within the meta-analysis. The test yielded no evidence of significant publication bias (P > 0.05), reinforcing the credibility of our meta-analytic results.

## 4 Discussion

The surgical management of NMIBC remains a cornerstone of urological oncology, with the primary goal being complete tumor resection while preserving bladder integrity. This systematic review and meta-analysis critically evaluate the efficacy of two prominent surgical techniques: Holmium Laser and Plasma Techniques. These modalities represent advanced surgical interventions that have evolved to improve patient outcomes and minimize procedural morbidity. The Holmium Laser, renowned for its precision, offers a minimally invasive approach that aligns with the current shift towards organ-sparing and function-preserving cancer treatments (17, 18). Its efficacy is not solely contingent upon tumor ablation but also encompasses the ability to minimize collateral damage, a crucial factor in maintaining bladder function post-surgery. Conversely, Plasma Techniques, leveraging the unique properties of ionized gas to ablate tissue, present a different set of advantages and limitations. As an emerging technology, Plasma Techniques have shown promise in providing effective tumor control with potentially reduced bleeding and shorter hospital stays (19, 20). Our meta-analysis scrutinizes three pivotal aspects of bladder cancer surgery: surgical duration, the incidence of bladder perforation, and postoperative tumor recurrence rates. These facets are paramount in determining the efficacy and safety of Holmium Laser and Plasma techniques in treating non-muscle invasive bladder cancer.

The lack of a significant difference in surgical duration between the Holmium Laser and Plasma groups highlights an essential consideration in surgical planning. Given the similarity in operative time, the decision-making process may rely more heavily on other factors such as equipment availability, surgeon expertise, and cost-effectiveness. The observed heterogeneity could be attributed to varying surgeon experiences, differences in equipment used, or the complexity of cases, which were not always standardized across the studies (21). These variables can have a profound impact on operative time, irrespective of the surgical method employed.

The notable reduction in bladder perforation rates with the Holmium Laser technique warrants attention. The precision of the Holmium Laser, with its ability to deliver energy in a highly controlled manner, may account for this finding. The laser’s ability to cut and coagulate tissues simultaneously allows for meticulous dissection with minimal collateral damage, reducing the risk of inadvertent bladder perforation. In contrast, Plasma techniques, while effective, may not offer the same level of precision, potentially leading to higher perforation rates. This finding is particularly relevant for surgical teams as they assess the risk-to-benefit ratio of the available surgical options. Perhaps the most clinically significant finding is the lower short-term recurrence rate of bladder tumors with the Holmium Laser technique (22, 23). The laser’s precise ablation may contribute to more complete tumor removal, leaving fewer residual tumor cells that could potentially cause recurrence. Additionally, the thermal effect of the Holmium Laser may induce a beneficial inflammatory response that could contribute to destroying microscopic tumor cells left after resection. This reduction in early recurrence is crucial, as it can decrease the need for subsequent interventions, reduce overall treatment costs, and improve patient quality of life (24).

The substantial heterogeneity observed in surgical duration (I² = 92.8%) may reflect several underlying differences across the included trials. First, tumor characteristics (size, location, and number) can directly affect resection complexity and therefore operative time; although some studies reported average tumor diameters, others did not, making it difficult to standardize this variable. Second, variations in surgeon experience and proficiency with Holmium‐laser or plasma devices could influence resection speed. For example, operators more familiar with one modality might complete resections more quickly, whereas less‐experienced surgeons may have longer learning curves. Third, the specific models and settings of the laser or plasmakinetic equipment (e.g., power output, fiber type, resection loop design) differed between trials and may have impacted cutting efficiency or hemostasis. Finally, operative‐time definitions were not uniform: some studies measured from cystoscope insertion to removal, while others excluded anesthesia induction or specimen retrieval. Although we used a random‐effects model to account for between‐study variability, readers should interpret the lack of a statistically significant difference in operative time with caution. In practice, individual surgeon familiarity and institutional protocols may influence whether one technique is faster in a given setting.

Hu et al.’s study (25) evaluated the efficacy and molecular characteristics of neoadjuvant RC48-ADC combined with immunotherapy in cisplatin-ineligible MIBC patients, identifying HER2 and HSPA1A expression in the C3 subcluster as potential predictive biomarkers. Their work underscores the growing importance of molecular stratification in guiding therapeutic decisions, particularly in advanced bladder cancer. Although their study addresses a different disease stage, its insights into tumor heterogeneity complement our findings by emphasizing the need for individualized treatment strategies in bladder cancer overall. Similarly, Hu et al.’s study (26) compared neoadjuvant immunotherapy, chemotherapy, and combination therapy in MIBC, highlighting the efficacy of combination regimens and proposing a pretreatment efficacy prediction model to inform therapy selection. Their study reinforces the value of multidisciplinary approaches and tailored treatment based on patient characteristics. Our meta-analysis focuses on NMIBC management, evaluating surgical modality outcomes rather than systemic therapies. Our pooled evidence from randomized controlled trials provides robust support for the superior safety profile and reduced recurrence rate associated with the holmium laser compared to plasma techniques. Together, these studies collectively underscore the critical importance of evidence-based, patient-centered approaches in managing bladder cancer across its disease spectrum. Our analysis complements emerging data in MIBC by filling a crucial gap in early-stage disease management, where optimizing surgical technique remains central to disease control, and may ultimately inform integrated, multimodal treatment strategies in the future.

Several limitations must be acknowledged. The relatively small number of included RCTs and limited sample sizes may constrain the generalizability of our findings. The included studies predominantly originated from specific geographic regions, potentially limiting applicability to broader patient populations and diverse clinical practices. Variations in surgeon expertise, follow-up duration, postoperative management, and outcome definitions introduce potential clinical heterogeneity. Some studies lacked detailed reporting on tumor grade, stage, and multifocality, which are critical predictors of recurrence and progression. Long-term oncologic outcomes, including progression to muscle-invasive disease and cancer-specific survival, were not consistently reported, precluding comprehensive assessment of oncologic durability. While our study demonstrates a significant reduction in short-term recurrence rates with holmium laser resection, the absence of mechanistic or translational research precludes definitive understanding of the biological rationale underlying these findings. Moreover, the substantial heterogeneity observed in operative-time analysis and the absence of a statistically significant time advantage for either technique further limit the robustness of conclusions regarding surgical efficiency. Future large-scale, multicenter prospective studies with extended follow-up are warranted to evaluate progression rates, survival outcomes, and quality-of-life metrics. Additionally, incorporating molecular and histopathological analyses, as well as assessing cost-effectiveness and learning curves, will enhance understanding of tumor biology and support the development of personalized surgical strategies for NMIBC.

## 5 Conclusions

In conclusion, while there is no significant difference in surgical duration when comparing Holmium Laser to Plasma Techniques, the Holmium Laser stands out for its ability to reduce intraoperative complications and lower the rate of postoperative tumor recurrence. This establishes the Holmium Laser as a safe, reliable, and effective surgical modality for the treatment of non-muscle invasive bladder cancer.

## Data Availability

The raw data supporting the conclusions of this article will be made available by the authors, without undue reservation.
